# Social Reward Questionnaire (SRQ): development and validation

**DOI:** 10.3389/fpsyg.2014.00201

**Published:** 2014-03-11

**Authors:** Lucy Foulkes, Essi Viding, Eamon McCrory, Craig S. Neumann

**Affiliations:** ^1^Developmental Risk and Resilience Unit, Division of Psychology and Language Sciences, University College LondonLondon, UK; ^2^Department of Psychology, University of North TexasTexas, TX, USA

**Keywords:** social reward, social motivation, social goals, dark triad, exploratory factor analysis, confirmatory factor analysis, scale development

## Abstract

Human beings seek out social interactions as a source of reward. To date, there have been limited attempts to identify different forms of social reward, and little is known about how the value of social rewards might vary between individuals. This study aimed to address both these issues by developing the Social Reward Questionnaire (SRQ), a measure of individual differences in the value of different social rewards. Exploratory factor analysis (EFA) was run on an initial set of 75 items (*N* = 305). Based on this analysis, confirmatory factor analysis (CFA) was then conducted on a second sample (*N* = 505) with a refined 23-item scale. This analysis was used to test a six-factor structure, which resulted in good model fit (CFI = 0.96, RSMEA = 0.07). The factors represent six subscales of social reward defined as follows: Admiration; Negative Social Potency; Passivity; Prosocial Interactions; Sexual Reward; and Sociability. All subscales demonstrated good test-retest reliability and internal consistency. Each subscale also showed a distinct pattern of associations with external correlates measuring personality traits, attitudes, and goals, thus demonstrating construct validity. Taken together, the findings suggest that the SRQ is a reliable, valid measure that can be used to assess individual differences in the value experienced from different social rewards.

## Introduction

Social stimuli are typically rewarding. For example, viewing static images of smiling faces results in increased activation in the striatum, part of the brain's reward network (Spreckelmeyer et al., 2009; Rademacher et al., 2013). More complex social experiences, such as sharing with a friend or being liked, are also found to activate the brain's reward network and are subjectively rated as enjoyable (sharing: Fareri et al., 2012; being liked: Izuma et al., 2008). Indeed, an absence or reduction in the reward value of social relationships is often associated with psychopathology. For example, social anhedonia is associated with depression (Blanchard et al., 2001; Forbes, 2009) and a reduced responsiveness to some social rewards is seen in autism (Dawson et al., 1998; Zeeland et al., 2010). It is therefore well established that social interactions are a source of reward for typically developing individuals, and that atypical social reward processing can be associated with clinical disorder.

More broadly, research with other types of rewards has found that individual differences in responsiveness to reward stimuli are predictive of individual differences in behavior toward those stimuli. For example, one study found that higher levels of trait reward sensitivity positively predicted overeating behavior, which in turn predicted a higher Body Mass Index (Davis et al., 2007). Heightened sensitivity to reward has also been found to predict alcohol misuse (Loxton and Dawe, 2001). By extension, understanding individual differences in the value of different social rewards may provide a useful clue to typical and dysfunctional social behavior.

However, experimental studies that measure social reward tend to use only one type of stimuli or experience to represent social reward. In general, the term *social reward* is used somewhat loosely across studies and typically denotes any social stimuli or interaction that participants appear to experience as rewarding/pleasurable. These issues preclude a fuller understanding of what social reward is and the range of social stimuli/experiences that elicit such reward.

To our knowledge, the only existing attempt to catalog different types of social rewards was made by Buss (1983), who defined a wide spectrum of social rewards from very basic (e.g., the presence of others) to more complex (e.g., the opportunity to self-disclose) and also predicted which personality traits may be associated with the value of different social rewards. Unfortunately, however, Buss's (1983) taxonomy of rewards was not empirically evaluated.

Empirically-driven categorizations of social goals may provide useful clues to the structure of social reward. Social goals can be defined as cognitive representations of desired social outcomes (McCollum, 2009) and one factor analysis study resulted in a seven-factor structure of social goals defined as follows: social responsibility and concern; social attractiveness; power; intimacy and interpersonal play; receiving assistance; belongingness; and giving (McCollum, 2009). Other studies have defined social goals in terms of the interpersonal circumplex (dominance, submissiveness, warmth, and hostility; Hill, 1987; Dryer and Horowitz, 1997), compared approach and avoidance goals (Gable, 2006) or based categorizations on video-taped observation of social interactions (Melnick and Hinshaw, 1996). These social goal categorizations are relevant to social reward, as goals are influenced by reward value (Elliot, 1999). However, these constructs are not equivalent to social reward, as measuring long-term goals does not necessarily measure the hedonic value of experiences (Ryan and Deci, 2001). For example, an individual could report a social goal to be fair to others, but does not necessary *enjoy* being fair. An outstanding challenge, therefore, is to identify and empirically evaluate a set of social rewards.

The current study aimed to create a questionnaire that both categorizes different types of social reward and measures individual differences in the degree to which each reward is valued. Questionnaire items were generated after reviewing papers that either explicitly discussed social reward or that assessed related social constructs (e.g., social goals). This initial questionnaire was completed by a first sample of participants. Exploratory factor analysis (EFA; Marsh et al., 2010) was used to identify the latent structure of the item set and to reduce its length, creating the Social Reward Questionnaire (SRQ). A second sample of participants then completed this refined questionnaire and a confirmatory factor analysis (CFA) was conducted to rigorously test the model generated via EFA. Participants in the second sample also completed a set of other questionnaires to assess the construct validity of the SRQ, and a subset of these participants completed the SRQ again 10–14 days later in order to assess test-retest reliability.

## Materials and methods

### Questionnaire development

As a starting point for item generation, theoretical and empirical literature discussing social reward and related constructs (e.g., social goals) were reviewed. The following conceptualizations and instruments were reviewed to identify a wide range of potential social rewards: Buss's (1983) theoretical taxonomy of social rewards, the Interpersonal Goal Inventory (Dryer and Horowitz, 1997), the resource theory of social exchange (Foa and Foa, 1980, 2012), approach and avoidance social motives and goals (Gable, 2006), social subscales of the Aspiration Index (Grouzet et al., 2005), the Interpersonal Orientation Scale (Hill, 1987), an adolescent Social Goals Questionnaire (Jarvinen and Nicholls, 1996), the Circumplex Scales of Interpersonal Values (Locke, 2000), McCollum's (2009) conceptualization of social goals, a taxonomy of children's social goals (Melnick and Hinshaw, 1996), the Short Sadistic Impulse Scale (O'Meara et al., 2011) and social items from the Snaith-Hamilton Pleasure Scale (Snaith et al., 1995).

Following this process, 19 potential types of social rewards were identified: affiliation/intimacy, aggression, being admired, being accepted/belonging, being sexually attractive, being socially responsible, competing with others, cruelty, dominance, having fun with others, instrumental gain, leadership, manipulation/coercion, nurturance/helping others, popularity, receiving assistance/care, sensation seeking with others, sexual reward, and submissiveness. It is important to note that the aim of this stage was to generate a wide range of social rewards, without presuming that the types of rewards identified would correspond to the actual factor structure of social reward.

Questionnaire items were then created to reflect the content of this wide array of social rewards. To ensure that items examined the hedonic value of each reward, all of the items began with the phrase “I enjoy” (Snaith et al., 1995). For example, the reward value of fairness was assessed with the statement “I enjoy being fair.” This phase generated a total of 123 items (five to nine items for each proposed type of social reward).

A panel of eight graduate-level psychology researchers with expertise in reward processing, social processing and/or social neuroscience were shown all 123 items grouped into the proposed types of social reward. Within each item group, the panel members were asked to score each item from 1 to 10 on how well it represented that proposed social reward (1 = Very badly to 10 = Very well). Each panel member worked independently. Within each group, the three to six items with the highest total scores were retained. The variance of the raters' scores for the retained items was low (mean *SD* = 1.01), indicating that there was high agreement of the best items. This process resulted in a total of 75 items, and the order was then randomized to create the pilot questionnaire. The category *sensation seeking with others* was dropped altogether in response to concerns from the panel about the clarity of this category.

A seven-point response scale (1 = Strongly disagree to 7 = Strongly agree) was chosen in order to sensitively capture differences in responses. Instructions were as follows: “*Here is a list of statements about what you enjoy when you interact with other people. The statements refer to all people in your life, e.g., friends, partners, family, colleagues or people you have just met. Consider how well each statement relates to you and indicate your answer from 1 to 7. NOTE: If there is something you have never experienced, imagine how much you* would *enjoy it*.”

### Sample 1: exploratory factor analysis (EFA)

#### Participants

Participants were recruited via Amazon's Mechanical Turk (MTurk; www.mturk.com), a crowd-sourcing website. MTurk is an international online platform that allows researchers to post tasks or questionnaires that participants complete in return for payment. MTurk is increasingly being used as a means of accessing experimental participants and conducting comprehensive surveys of general population samples (Buhrmester et al., 2011; Mathieu et al., 2013). In the current study, participants signed up via MTurk and were then directed to the online survey software LimeSurvey (www.limesurvey.org) to complete the questionnaire. The questionnaire took approximately 10 min to complete and participants were compensated $0.40 for their time.

The 75-item pilot questionnaire was completed 320 times. Ten responses were removed because the same participant had completed the questionnaire twice (the second response was removed). A further five participants were excluded due to lack of variability in responses (e.g., one answered “Neither agree nor disagree” to 74 of the 75 items). This left a total of 305 participants in Sample 1.

Participants (151 females; 127 males; 27 undisclosed gender) were aged 18–70 years old (mean = 33.9, *SD* = 12.1). The highest completed education level of the sample was as follows: 38.4% Bachelor's degree, 19.0% College, 17.7% Postgraduate degree and 16.1% senior school (undisclosed for 8.85%).

#### Data analysis procedure

To explore the latent structure of the social reward item set, a series of EFAs were run using Mplus (Muthen and Muthen, 2010). Due to the ordinal nature of the items, the items were treated as polytomous and analyzed using polychoric correlations via the mean and variance adjusted weighted least squares (WLSMV) estimation procedure (Muthen and Muthen, 2010).

This analysis provided model fit statistics, which allowed the relative strengths of exploratory-derived factor solutions to be assessed without the need for specifying the factor structure in advance (Mora et al., 2011; Marsh et al., 2013).

As recommended by Hu and Bentler (1999), we used a two-index strategy to assess model fit: the incremental Comparative Fit Index (CFI) and the Root Mean Square Error of Approximation (RMSEA), an absolute fit index. Hu and Bentler (1999) suggested that a CFI of 0.95 or higher and an RMSEA of 0.08 or lower were indicative of good model fit. However, these fit indices may be too strict and can be questioned in terms of both practical and substantive significance (Marsh et al., 2004; Hopwood and Donnellan, 2010). We therefore adopted the traditional CFI of 0.90 or above and RMSEA of 0.08 or below (West et al., 2012) as indicative of acceptable model fit.

#### Results from EFA

There were no missing data, as the questionnaire was programmed in such a way that all items required a response. The EFA identified nine factors with an eigenvalue greater than 1.5, which suggested a nine-factor structure. The nine-factor solution was also the most parsimonious solution that was associated with good model fit (CFI = 0.96, RMSEA = 0.04). However, two factors were weak: one factor contained only two items, both of which crossloaded >0.50 onto other factors; the other contained only three items, two of which crossloaded >0.50 onto other factors. These two factors were dropped from the solution. A third factor had two items that loaded very strongly (>0.80) and four weak items (i.e., they had a secondary loading that was >0.40 and/or <0.15 difference between the primary and secondary loading). The two strong items correlated very highly with each other (*r* = 0.83, *p* < 0.001), suggesting that this factor may be a very narrow construct. For this reason, this factor was also dropped from the solution. The remaining six factors all had at least three items with loadings >0.46. These six factors were defined as follows: Admiration, Negative Social Potency, Passivity, Prosocial Interactions, Sexual Relationships, and Sociability (see Table 1).

**Table 1:** **Description of factors identified via EFA**.

**Name of factor**	**Description**	**Example item**
Admiration	Being flattered, liked and gaining positive attention	*“I enjoy achieving recognition from others”*
Negative Social Potency	Being cruel, callous and using others for personal gains	*“I enjoy embarrassing others”*
Passivity	Giving others control and allowing them to make decisions	*“I enjoy following someone else's rules”*
Prosocial Interactions	Having kind, reciprocal relationships	*“I enjoy treating others fairly”*
Sexual Relationships	Having frequent sexual experiences	*“I enjoy having an active sex life”*
Sociability	Engaging in group interactions	*“I enjoy going to parties”*

#### Item reduction

Several further steps were taken to reduce the length of the questionnaire (Worthington and Whittaker, 2006). All decisions were based on the results from the original EFA. Firstly, items that did not load strongly onto any of the six factors were removed (10 items; all loaded <0.40 on all factors). Secondly, any item that crossloaded onto two or more factors was removed (12 items; all loaded >0.40 on at least two factors). Finally, in order to create a succinct scale, only the best items from each factor were selected for retention (on the basis of meaningfully representing the factor, having the highest loading, and/or the lowest crossloading; Worthington and Whittaker, 2006). This resulted in a 23-item scale with six subscales.

To explore the strength of the proposed 23-item scale before collecting data from a new sample, a CFA was run with Sample 1 on the chosen 23 items. The model fit supported the proposed six-factor structure (CFI = 0.95, RMSEA = 0.06).

### Sample 2: confirmatory factor analysis (CFA) and construct validation

Sample 2, the replication sample, was collected in the second phase to confirm the structure, validity and reliability of the 23-item SRQ.

The replication sample (*N* = 505) was adequate for testing a model consisting of 61 parameters (i.e., 23 factor loadings, 23 error variances, 15 factor correlations). Specifically, the 23-item model approximates an 8:1 subjects-to-parameters ratio, approaching the 10:1 ratio recommended by Bentler and Chou (1987). We note that Bentler and Chou (1987) suggested that this ratio could go as low as 5:1 if the items have good measurement characteristics. Given that the SRQ items were derived from established measures, it is reasonable to propose that they have robust statistical properties, and therefore the 8:1 ratio was deemed adequate for the replication CFA. The analyses that follow support this proposal.

Firstly, CFA was conducted on the 23-item SRQ. We again used the WLSMV estimation procedure as recommended for analysis of ordinal data (Muthen and Muthen, 2010). Secondly, participants in Sample 2 also completed a set of established questionnaires measuring personality traits, attitudes, and goals to confirm the construct validity of the SRQ. Finally, a subset of participants from Sample 2 (*N* = 45) completed the SRQ for a second time in order to measure test-retest reliability. All correlational analyses were Pearson zero-order correlations, conducted using IBM SPSS Statistics 20 for Windows.

#### Participants

Amazon's MTurk platform was used again to recruit 529 participants. Participants were excluded for providing obviously repetitive answers (*N* = 5), or for completing the questionnaire battery twice (second attempt excluded; *N* = 19). The final sample therefore consisted of 505 participants (270 males, 235 females) aged 18–79 years (mean 34.0, SD 12.2). The ethnicity of the sample was as follows: 72.3% White, 11.1% South Asian, 6.1% Black, 2.8% Hispanic, 2.0% East Asian and 5.7% Mixed/Other. The highest completed education level of the sample was as follows: 38.2% Bachelor's degree, 30.9% Senior/high school, 18.8% College, 12.1% Postgraduate degree. The questionnaires took approximately 10 min to complete and participants were paid $0.40 for their time.

#### Measures

In addition to the SRQ, participants completed the following questionnaires for the purposes of construct validity:

#### Dirty dozen (Jonason and Webster, 2010)

This is a 12-item scale with three subscales, each measuring one component of the “Dark Triad”: Machiavellianism, narcissism and psychopathy (Paulhus and Williams, 2002). Respondents are asked to indicate how much they agree with each item on a 1–5 scale (1 = Not at all, 5 = Very much).

We hypothesized that SRQ Negative Social Potency would be positively associated with all Dark Triad subscales and SRQ Prosocial would be negatively associated with them. We also hypothesized that SRQ Admiration would be positively associated with narcissism.

#### Interpersonal goal inventory (Dryer and Horowitz, 1997)

This is a 32-item questionnaire that measures the importance of different interpersonal goals. It consists of eight subscales that reflect the four extremes of the interpersonal circumplex (e.g., Wiggins, 1979): Dominance, Submissiveness, Friendliness and Hostility, and the octants between them (Dominant/friendly, Dominant/hostile, Submissive/friendly, Submissive/hostile). Each item begins “It would be important for me to … ” and responses are given on a 0–4 scale (0 = No, definitely not, 4 = Yes, definitely).

We hypothesized that SRQ Passivity would be positively correlated with Submissiveness and negatively correlated with Dominance. We also hypothesized that SRQ Prosocial would be positively associated with Friendliness. Finally, we hypothesized that SRQ Negative Social Potency would be positively associated with Hostility^1^.

#### Ten-item personality inventory (Gosling et al., 2003)

This is a 10-item scale that measures the “Big Five” personality traits (agreeableness, conscientiousness, extraversion, neuroticism and openness to experience; e.g., Costa and McCrae, 1992). All items begin “I see myself as” and are followed by two descriptive items such as “Anxious, easily upset.” Responses are given on a 1–7 scale (1 = Disagree strongly, 7 = Agree strongly).

We hypothesized that SRQ Prosocial would be positively associated with agreeableness and conscientiousness. We also hypothesized that SRQ Negative Social Potency would be negatively correlated with these traits. Finally, we hypothesized that SRQ Sociability would be positively correlated with extraversion.

#### Revised sociosexual orientation inventory (Penke and Asendorpf, 2008)

This is a nine-item scale with subscales indicating three aspects of sexual promiscuity: behavior, attitude and desire. Responses are given on nine-point scales.

We hypothesized that SRQ Sexual Relationships would be positively correlated with all three subscales.

## Results

There were no missing data. The six-factor model developed from Sample 1 achieved good fit using the data from the replication sample, Sample 2 [χ^2^_(215)_ = 747.77, *p* < 0.001; CFI = 0.96; RMSEA = 0.07, 90% CI = 0.07–0.08]. Factor loadings were in the range 0.62–0.92 (mean = 0.79, *SD* = 0.08) and are shown in Table 2.

**Table 2:** **Standardized factor loadings from the six-factor CFA**.

**Factor**	**Loading**	**Item number**
Admiration	0.82	1
	0.69	7
	0.80	11
	0.76	18
Negative Social Potency	0.80	3
	0.77	5
	0.85	8
	0.85	14
	0.92	17
Passivity	0.79	12
	0.62	21
	0.90	23
Prosocial Interactions	0.81	2
	0.72	6
	0.74	16
	0.76	19
	0.84	22
Sexual Relationships	0.90	9
	0.78	13
	0.86	20
Sociability	0.71	4
	0.62	10
	0.90	15

### Reliability

Correlations, Cronbach alphas and mean inter-item correlations (MICs) of manifest subscale scores are shown in Table 3. Cronbach alphas for all subscales were good and demonstrate that they are internally consistent (mean = 0.82, *SD* = 0.04; range = 0.77–0.87). With regard to scale homogeneity, the MICs were acceptable (mean = 0.56, *SD* = 0.05; range = 0.51–0.65) for subscales measuring relatively narrow constructs (Clark and Watson, 1995), as was our intention. This further suggests that the items reflect unidimensional measures of their respective subscales.

**Table 3:** **Correlations, descriptives (mean and SD), Cronbach alphas and mean interitem correlations (MIC) for manifest factor totals in Sample 2 (*N* = 505)**.

	**1**	**2**	**3**	**4**	**5**	**6**	**Mean^+^ (*SD*)**	**MIC**
1. Admiration	0.82						5.09 (1.14)	0.53
2. Neg Soc Pot	−0.03	0.87					2.04 (1.09)	0.58
3. Passivity	−0.02	0.32^**^	0.78				3.13 (1.27)	0.54
4. Prosocial	0.35^**^	−0.56^**^	−0.09^*^	0.84			5.98 (0.85)	0.51
5. Sexual	0.34^**^	0.00	−0.01	0.22^**^	0.84		5.06 (1.53)	0.65
6. Sociability	0.53^**^	0.02	0.03	0.25^**^	0.32^**^	0.77	4.61 (1.39)	0.53

Factor correlations with p < 0.05 are shown in bold;

** *p < 0.01*,

* p < 0.05; Cronbach alphas appear on the diagonal;

+ *Mean item score in each factor*.

### Test-retest reliability

In order to measure test-retest reliability of the SRQ, 45 participants from Sample 2 completed the SRQ twice. (Participants who had most recently taken part (*N* = 100) were invited to complete the questionnaire a second time for a small fee; 45 participants responded). The time between the two testing points ranged from 10 to 14 days (mean = 12.0, *SD* = 1.3).

Pearson correlations between each subscale at the two time points were good (mean = 0.80, *SD* = 0.06, all *p* < 0.001; see Table 4). This indicates the stability of questionnaire responses across time.

**Table 4:** **Test-retest reliability: Pearson correlations between factor subtotal scores at Time 1 and Time 2 (mean time interval = 12 days)**.

**Subscale**	**Correlations between SRQ subscales at Time 1 and Time 2**
Admiration	0.69
Negative Social Potency	0.88
Passivity	0.83
Prosocial Interactions	0.78
Sexual Relationships	0.82
Sociability	0.78

*All p < 0.001*.

### Construct validity

Pearson correlational analyses were used to explore the pattern of associations between the six SRQ subscales and other related measures. Benjamini and Hochberg False Discovery Rate (Benjamini and Hochberg, 1995) was used to control for the probability of making a Type I error on multiple comparisons, and corrected *p*-values are presented in Table 5.

**Table 5:** **Pearson correlations between SRQ subscales and external measures**.

	**SRQ subscale**					
	**Admiration**	**Negative Social Potency**	**Passivity**	**Prosocial Interactions**	**Sexual Relationships**	**Sociability**
**DARK TRIAD**						
Machiavellianism	0.05	0.62^**^	0.12^*^	−0.34^**^	0.11^*^	0.08
Narcissism	0.42^**^	0.31^**^	0.07	−0.10^*^	0.16^**^	0.32^**^
Psychopathy	−0.04	0.59^**^	0.13^*^	−0.41^**^	0.08	−0.07
**INTERPERSONAL GOALS**						
Dominance	0.32^**^	−0.24^**^	−0.25^**^	0.44^**^	0.23^**^	0.19^**^
Friendliness	0.16^**^	−0.41^**^	−0.03	0.52^**^	0.16^**^	0.15^**^
Hostility	0.20^**^	0.31^**^	−0.05	−0.19^**^	−0.04	0.04
Submissiveness	0.05	−0.20^**^	0.12^*^	0.28^**^	0.03	−0.04
**SOCIOSEXUAL ORIENTATION**						
Attitude	0.16^**^	0.07	−0.06	−0.01	0.53^**^	0.22^**^
Behavior	0.05	0.16^**^	−0.05	−0.08	0.33^**^	0.24^**^
Desire	0.11^*^	0.26^**^	0.02	−0.11^*^	0.47^**^	0.13^**^
**PERSONALITY**						
Agreeableness	0.05	−0.48^**^	−0.02	0.44^**^	−0.00	0.10^*^
Conscientiousness	0.08	−0.39^**^	−0.15^*^	0.34^**^	0.02	0.04
Emotionality	0.05	−0.19^**^	−0.19^**^	0.15^**^	0.06	0.17^**^
Extraversion	0.19^**^	−0.03	−0.09	0.13^**^	0.11^*^	0.37^**^
Openness	0.29^**^	0.19^**^	−0.14^**^	0.33^**^	0.28^**^	0.28^**^

*Correlations of p < 0.05 after correcting for multiple comparisons are in bold*.

* p < 0.05

** *p < 0.01*.

The subscales of the SRQ showed expected associations with the external correlates, providing evidence that each subscale is measuring a relatively distinct social reward. SRQ Admiration was positively correlated with narcissism, the attitude and desire subscales of sociosexual orientation, extraversion, and openness. SRQ Negative Social Potency was positively associated with all three Dark Triad traits, hostility, sexual behavior and desire, and openness. SRQ Passivity was positively correlated with submissiveness, Machiavellianism, and psychopathy, and negatively associated with dominance, conscientiousness, emotionality, and openness. SRQ Prosocial Interactions was positively associated with dominance, friendliness and all personality subscales, and negatively associated with all Dark Triad traits, hostility, and sexual desire. SRQ Sexual Relationships was positively associated with Machiavellianism, narcissism, all sociosexual orientation subscales, extraversion and openness. Finally, SRQ Sociability was positively correlated with narcissism, dominance, friendliness, all sociosexual orientation subscales and all personality subscales except conscientiousness.

## Discussion

The 23-item SRQ is a comprehensive measure of individual differences in the value of social rewards. Using EFA and CFA, we identified six subscales of the SRQ that equate to six social reward domains: Admiration; Negative Social Potency; Passivity; Prosocial Interactions; Sexual Relationships; and Sociability. The results indicate that the SRQ has a clear factor structure and strong psychometric properties.

Different subscales of the SRQ showed distinct associations with external correlates, which provides support for the meaning of each scale and suggests that the subscales capture different aspects of social reward. Discussion of every association between the different subscales and external correlates is beyond the scope of this paper, but here we highlight some key findings. For example, SRQ Admiration was positively correlated with narcissism, a cluster of traits defined by self-love (Jones and Paulhus, 2010). SRQ Negative Social Potency was positively correlated with all Dark Triad traits and negatively correlated with friendliness, agreeableness and conscientiousness, suggesting this subscale does indeed capture enjoyment of callous and inconsiderate behavior toward others. SRQ Prosocial Interactions showed the mirror opposite pattern of associations to SRQ Negative Social Potency, although it is important to note that the association between these two factors, while moderately strong (*r* = −0.56, *p* < 0.001), does not indicate that they are two extremes of the same concept. SRQ Passivity was positively associated with submissiveness and negatively associated with dominance as predicted, but was unexpectedly positively correlated with narcissism and psychopathy and negatively with conscientiousness, emotionality and openness. We are not entirely sure how to interpret these associations, but it may be that SRQ Passivity does not measure the enjoyment of mere submissiveness but rather a social laziness, a desire to be a “free rider” and let others do the work. Finally, SRQ Sexual Relationships showed the expected correlations with sociosexual orientation, and SRQ Sociability was correlated with extraversion as expected.

This pattern of associations with external correlates suggests the utility of the SRQ in understanding certain social behaviors. For example, the positive correlation between SRQ Negative Social Potency and all Dark Triad traits could provide an overlooked clue as to why people behave cruelly toward others: they enjoy it. Sadism is primarily the enjoyment of seeing others in physical pain (O'Meara et al., 2011), but pleasure from others' *psychological* pain, as measured by SRQ Negative Social Potency, could be a significant adjunct to this. This is an important avenue to explore when trying to understand antisocial behavior. In general, the relationship between social reward, personality and social behavior needs to be explored in future research.

Beyond understanding individual differences in typical populations, we suggest that the SRQ may have clinical utility. For example, a diminished experience of reward, including from social relationships, is symptomatic of depression (Blanchard et al., 2001). Secondly, atypical social reward may be relevant in a number of personality disorders, as indicated by associations in the current study between the SRQ subscales and Machiavellianism, narcissism and psychopathy. Finally, individuals with autism experience lower levels of reward from social stimuli and this may be a key feature of the condition (Social Motivation Hypothesis; Dawson et al., 1998; Zeeland et al., 2010). It would be important to accurately delineate the profile of attenuated and preserved social reward across these conditions. The SRQ may be helpful in this regard, but as a self-report measure should be interpreted with caution in individuals with autism, given the known difficulties with introspection in this group (e.g., Lombardo et al., 2010). Finally, there may also be interest in exploring gender or ethnicity differences in relation to social reward.

It is important to note limitations of the SRQ. Firstly, social reward is a complex construct; as a questionnaire, the SRQ will entail a degree of simplification that may obscure more nuanced aspects of the phenomenon. Secondly, this is the first study to empirically explore the underlying structure of social reward. It will be important for future studies to replicate the factor structure in other samples, and also to replicate the test-retest reliability with larger samples (Watson, 2004). Finally, there may be other aspects of social reward that are not explored with the SRQ, and which have yet to be accurately identified in the existing literature. However, the SRQ provides a promising basis to further empirically assess individual differences in social reward.

## Conclusion

The SRQ is the first measure of individual differences in the value of different types of social rewards. Using EFA and CFA, six social rewards were identified in the current study: Admiration, Negative Social Potency; Passivity; Prosocial Interactions; Sexual Relationships; and Sociability. These six social rewards were found to be robustly and differentially associated with a variety of self-reported personality traits, attitudes and goals. We propose that the SRQ is a valid, reliable measure that has value in the study of social reward.

### Conflict of interest statement

The authors declare that the research was conducted in the absence of any commercial or financial relationships that could be construed as a potential conflict of interest.

## Footnotes

^1^In the current study, we analyzed only the subscales representing the extremes of the circumplex (Dominance, Submissiveness, Friendliness, and Hostility) and not those representing the octants between them. This was for purposes of clarity and simplicity. Full analyses of all subscales are available on request from the first author).
